# Riluzole-induced apoptosis in osteosarcoma is mediated through Yes-associated protein upon phosphorylation by c-Abl Kinase

**DOI:** 10.1038/s41598-021-00439-8

**Published:** 2021-10-25

**Authors:** Marian Raghubir, Syeda M. Azeem, Rifat Hasnat, Chowdhury N. Rahman, Linda Wong, Salina Yan, Yu Qi Huang, Raquel Zhagui, Angelina Blyufer, Iffat Tariq, Cassey Tam, Sonam Lhamo, Lucas Cecilio, Yesmin Chowdhury, Shraddha ChandThakuri, Shahana S. Mahajan

**Affiliations:** 1 Department of Medical Laboratory Sciences, Hunter College, City University of New York, 425 East, 25th Street, New York, NY 10010 USA; 2 Ph.D. Program in Biochemistry, The Graduate Center of the City University of New York, New York, NY 10016 USA; 3 Ph.D. Program in Biology, The Graduate Center of City University of New York, New York, NY 10016 USA; 4 Brain Mind Research Institute, Weill Cornell Medical College, 413 East 69th Street, New York, NY 10021 USA

**Keywords:** Biochemistry, Biological techniques, Cancer, Cell biology, Drug discovery, Molecular biology

## Abstract

Our lab has previously demonstrated Riluzole to be an effective drug in inhibiting proliferation and inducing apoptosis in both human and mouse osteosarcoma. Yes-associated protein is a transcription co-activator, known to be involved in cell proliferation or apoptosis depending on its protein partner. In the present study we investigated the role of YAP in apoptosis in osteosarcoma, we hypothesized that YAP may be activated by Riluzole to induce apoptosis in osteosarcoma. By knocking down the expression of YAP, we have demonstrated that Riluzole failed to induce apoptosis in YAP deficient osteosarcoma cells. Riluzole caused translocation of YAP from the cytoplasm to the nucleus, indicating YAP’s role in apoptosis. Both Riluzole-induced phosphorylation of YAP at tyrosine 357 and Riluzole-induced apoptosis were blocked by inhibitors of c-Abl kinase. In addition, knockdown of c-Abl kinase prevented Riluzole-induced apoptosis in LM7 cells. We further demonstrated that Riluzole promoted interaction between YAP and p73, while c-Abl kinase inhibitors abolished the interaction. Subsequently, we demonstrated that Riluzole enhanced activity of the Bax promoter in a luciferase reporter assay and enhanced YAP/p73 binding on endogenous Bax promoter in a ChIP assay. Our data supports a novel mechanism in which Riluzole activates c-Abl kinase to regulate pro-apoptotic activity of YAP in osteosarcoma.

## Introduction

Osteosarcoma is a primary malignant bone tumor^1^, derived from mesenchymal cells that fail to differentiate into osteoblasts^2^. Metastasis of osteosarcoma is presented at the time of diagnosis in ~ 15–20% of cases^3,4^. The changes in the tumor microenvironment and the metastasis contribute to the failure of the conventional drug delivery approach^5–7^. A drug therapy approach would be more successful only if osteosarcoma cells are specifically targeted. One such drug is Riluzole, which is used in neurological diseases and shows some promise in cancer treatment, especially for cancers secreting glutamate.

Osteosarcomas have been shown to secrete glutamate and stimulate autocrine glutamate signaling for growth^8^. We have used human metastatic osteosarcoma, LM7 cells (derived from Saos-LM6 cells)^9^ to study the effect of Riluzole. We have previously demonstrated that LM7 cells secrete glutamate, and Riluzole blocks the secretion of glutamate, thereby inhibiting the autocrine effect on LM7 cells^8^. We have also demonstrated that LM7 cells express the metabotropic glutamate receptor, mGluR5, and knockdown of mGluR5 prevented the colony forming ability of LM7 cells^8^. Thus, Riluzole is an effective drug that inhibits cell proliferation and induces apoptosis in several types of cancers, however, the mechanism of action of Riluzole in osteosarcoma is not known.

Riluzole, was first shown to prevent glutamate secretion in brain slices^10^. Although the mechanism of action of Riluzole was not clear, it was later shown to block sodium channels as well as glutamate signaling^11,12^. The use of Riluzole in several neurological diseases such as Amyotrophic Lateral Sclerosis (ALS) and Parkinson’s Disease is well known^13^. However, Riluzole has been proven to have anti-cancer activity, therefore, Riluzole is being repurposed for cancer treatment. For instance, the treatment of triple negative breast cancer cells with Riluzole inhibits cell proliferation^14^. Importantly, Riluzole has been observed to reduce the growth of cancer cells in culture or in xenograft models for brain, skin, breast, pancreas, liver and prostate cancers^8,9,14–23^. In a clinical trial for melanoma patients, Riluzole decreased tumor size in a number of patients^22^. Furthermore, in a phase II trial in patients with advanced GRM1-positive melanoma, Riluzole showed some clinical benefits^17^. Thus Riluzole has shown promise as an anti-cancer agent in cancers of various tissue origin.

YAP, Yes-associated protein, is a potent growth promoter that is restrained by and is a downstream effector of the Hippo pathway regulating organ size, tissue homeostasis, stem cell self-renewal, cell proliferation and apoptosis^24–27^. Several G-protein coupled receptors (GPCRs) are found to be upstream activators of the Hippo pathway^28^. We have demonstrated that mGluR5, a GPCR, activates glutamate dependent growth signaling in osteosarcoma cells^8^. YAP is overexpressed in osteosarcoma and the knockdown of YAP resulted in tumor shrinkage in mice^29^. YAP is a co-activator and associates with a variety of transcription factors to induce the transcription of genes^30^. YAP induces the transcription of genes involved in cell cycle control^31–33^. However, upon DNA damage, YAP induces apoptosis^34,35^. Numerous studies support the conflicting functions of YAP as a tumor suppressor and as a tumor promoter, which seems to depend on specific cell type and the nature of the signal^30,36^. Phosphorylation of YAP at specific sites regulates its activity and outcome in cell proliferation or apoptosis^36^. YAP is phosphorylated at serine 127 by Akt/PKB or Lats1 and Lats2 (Large tumor suppressor)^34,37–39^. Lats1 and Lats2 are members of the Hippo pathway and inhibit oncogenic activity of YAP by sequestering YAP in the cytoplasm^38,40^. Moreover, Lats2 phosphorylates YAP at serine 397 to promote ubiquitin-mediated degradation of YAP^41^. c-Abl, a tyrosine kinase, is activated during DNA damage-induced apoptosis^42^. Interestingly, Lats2 inhibits the activity of c-Abl to prevent DNA damage-induced apoptosis^43^. During DNA damage, YAP binds to another transcription factor, p73, and this interaction is enhanced by YAP preventing ubiquitination of p73^44–46^. In addition, c-Abl phosphorylates p73 to stabilize p73 during DNA damage^42,47,48^. In this report we have examined the impact of the previously unrecognized p73/c-Abl axis on YAP dependent effects of Riluzole in osteosarcoma. We show that the regulation of YAP phosphorylation at specific sites by c-Abl appears to determine its role in apoptosis in osteosarcoma. Thus, we have determined a mechanism in which Riluzole recruits YAP to induce apoptosis in osteosarcoma.

## Results

### Knockdown of YAP reduces Riluzole induced apoptosis in osteosarcoma

**Figure 1:**
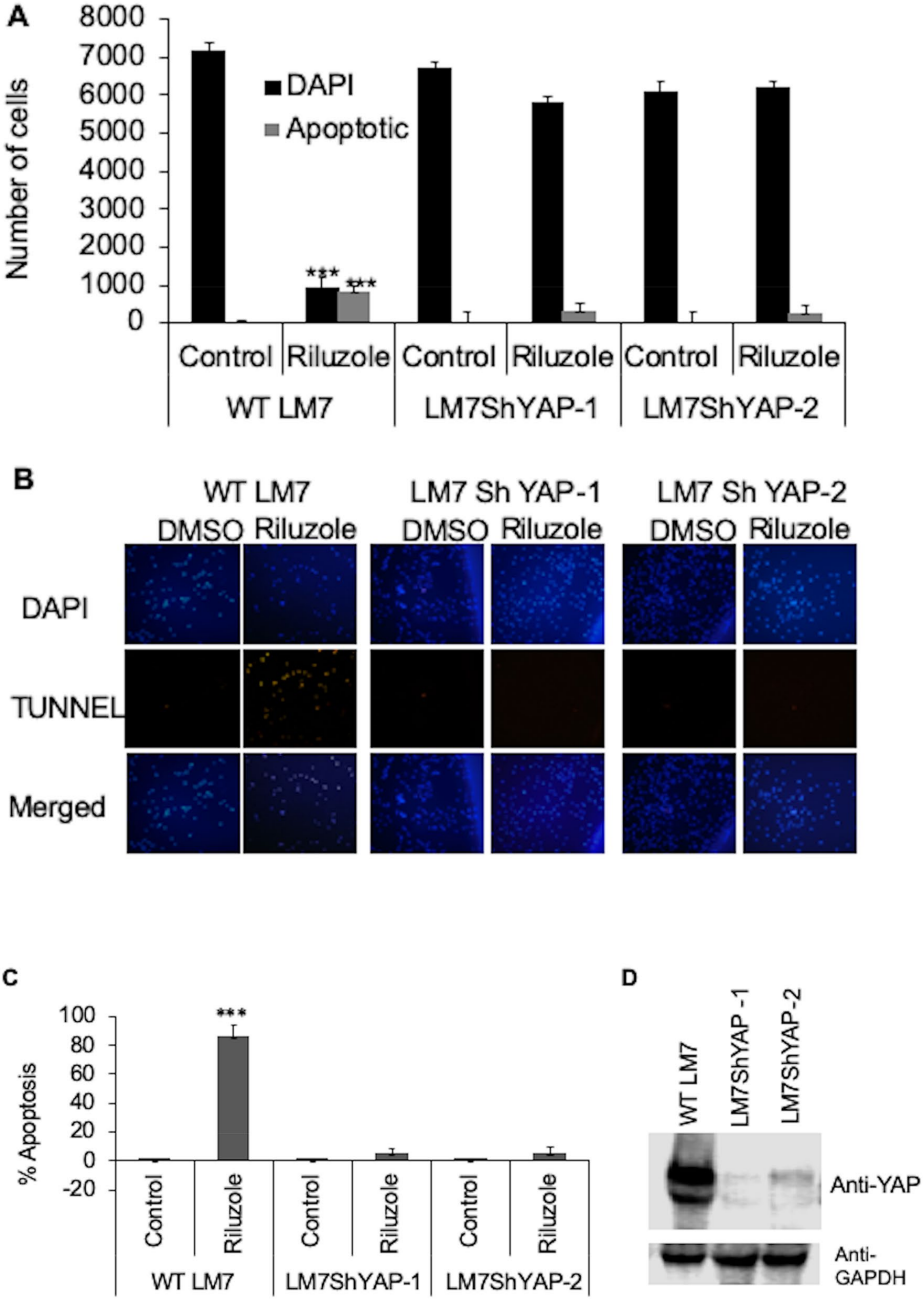
YAP knockdown decreases Riluzole induced apoptosis in LM7 cells. (**A**) Apoptosis in WT LM7 cells with control shRNA or LM7shYAP-1and LM7shYAP-2 with shYAP were treated with Riluzole (100 μM) or DMSO for control. DAPI positive and TUNEL positive cells were scored by fluorescence microscopy. Triplicate samples were analyzed for each treatment. The experiment was repeated three times, p value ***< 0.001. (**B**) Representative images of TUNEL assay are shown. (**C**) Percentage of apoptotic cells was analyzed based on the number of DAPI positive and TUNEL positive cells. (**D**) Western blot of YAP expression in WT LM7 cells and LM7shYAP cells using mouse anti-YAP antibody and reprobed with rabbit anti-GAPDH as a loading control.

To determine if YAP is a key protein involved in the execution of apoptosis by Riluzole, we have knocked down the expression of YAP using lentivirus expressing shRNA against YAP. Stable cell lines expressing shRNA for YAP or non-target shRNA as a control were tested in a TUNEL assay. Wild type LM7 containing non-target shRNA control showed apoptosis upon Riluzole treatment compared to DMSO treated control. The number of DAPI positive cells reduced significantly due to loss of cells from apoptosis. However, in stable cell lines LM7shYAP-1 lines and LM7shYAP-2 with YAP knockdown, Riluzole failed to induce apoptosis as seen by the number of DAPI positive cells, which are not significantly different from the DMSO control sample (Fig. 1A). The percentage of apoptosis was determined by quantifying the number of TUNEL positive apoptotic cells compared to the DAPI positive cells. The representative images of the TUNEL assay are shown (Fig. 1B). The results clearly show a highly significant decrease in apoptosis in LM7shYAP-1 and LM7shYAP-2 cells without YAP (Fig. 1C). Stable LM7shYAP-1 and LM7shYAP-2 cells showed a significantly reduced expression of YAP compared to WT with the control shRNA in western blots with anti-YAP antibody. GAPDH expression is shown as a loading control (Fig. 1D). The results provide evidence that YAP plays an important role in the induction of apoptosis by Riluzole in LM7 cells.

### Riluzole changes the localization of endogenously and exogenously expressed YAP from cytoplasmic to nuclear

**Figure 2:**
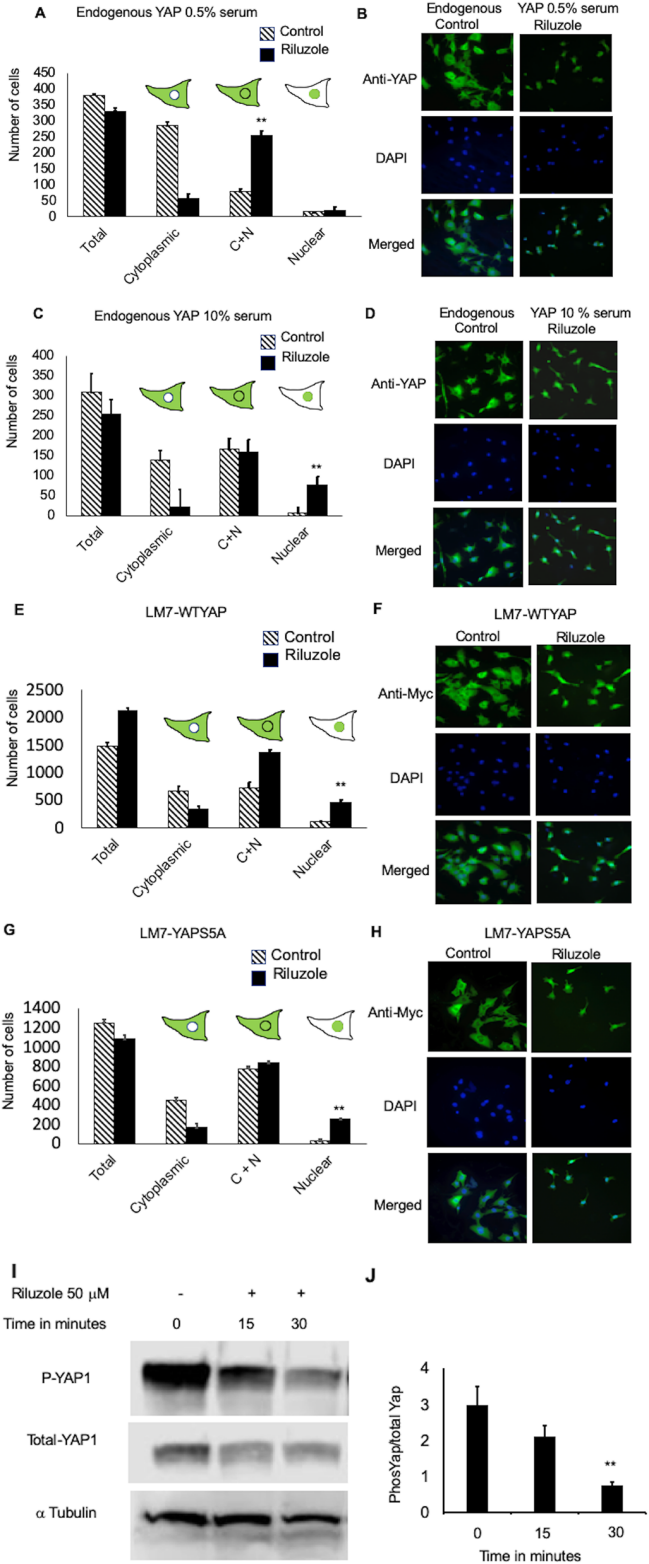
Riluzole alters subcellular localization of YAP. (**A**). Subcellular localization of endogenous YAP was measured in LM7 cells grown in 0.5% serum, p value < 0.05**. (**B**) Representative images for LM7 cells grown in 0.5% serum. (**C**) In 10% serum, p value < 0.05**. (**D**) Representative images for LM7 cells grown in 10% serum. (**E**) Subcellular localization of stably expressed Myc-tagged WT-YAP in LM7 cells, p value < 0.05**. (**F**) Representative images for Myc-tagged WT-YAP in LM7 cells. (**G**) YAP mutant (YAP S5A) (C + N = cytoplasmic + nuclear), p value < 0.05**. (**H**). Representative images of Myc-tagged YAP mutant (YAP S5A) LM7 cells. The cells were treated with DMSO or Riluzole (50 μM) for 1 h, fixed and stained with either anti-YAP (**A**–**D**) or anti-Myc (**E**–**H**) antibody. The samples were mounted in DAPI containing media. Fluorescence microscopy was performed on blinded samples and YAP localization was analyzed from triplicate samples in each four independent experiments. (**I**) Whole cell extracts were analyzed by western blot using either anti-phospho 127, anti-YAP or anti tubulin antibodies. (**J**) Intensity of the bands was measured by densitometry using LiCOR imaging system. The ratio of Phospho YAP to total YAP is represented. The experiment was repeated this experiment three times with triplicate samples in each trial, p value < 0.005**.

We have shown that Riluzole effectively induces apoptosis in human metastatic osteosarcoma and mouse osteosarcoma cell lines^8^. Phosphorylation of YAP at specific sites controls cytoplasmic versus nuclear localization of YAP thereby controlling YAP stability and transcriptional activation function^39–41^. To investigate the mechanism of action of Riluzole in apoptosis, we studied the effect of Riluzole on the cellular localization of endogenous YAP in LM7 cells. Low serum is known to induce apoptosis by translocation of YAP in HEK cells^39^. Therefore, we studied the effect of low (0.5%) or normal (10%) serum on the subcellular localization of YAP. In both 0.5% and 10% serum, Riluzole significantly shifted the localization of endogenous YAP from cytoplasm to nucleus as seen by the increase in cytoplasmic + nuclear or increase in nuclear localization (Fig. 2A,C). Thus, our data showed that Riluzole changed the localization of endogenous YAP in low and high serum growth conditions. We stably expressed Myc-tagged YAP using a lentivirus expression system in LM7 cells (LM7-WTYAP) and tested the effect of Riluzole on cellular localization of exogenous YAP. We performed localization experiments as described above using anti-Myc antibody and secondary antibody conjugated to Alexa-488. As expected, Riluzole changed the localization of Myc-tagged YAP from cytoplasm to nucleus (Fig. 2E).

### Riluzole affects the localization of YAP mutant (S5A) in a similar fashion to WT YAP

YAP induces the transcription of either pro-proliferating genes or pro-apoptotic genes depending on the phosphorylation of distinct sites^30,36^. For example, the phosphorylation of serine at S61, S109, S127, S164, and S397 is known to induce cell growth^38^. We used a lentivirus expressing Myc-tagged YAP mutant, YAPS5A (five serines at 61, 109, 127, 164, and 397 were changed to alanine) to make stable cell lines expressing YAPS5A. We then tested the effect of Riluzole on stable LM7 cell line expressing Myc-YAPS5A (LM7-YAPS5A) on cellular localization and found that Riluzole still induced the translocation of cytoplasmic Myc-YAPS5A to nucleus (Fig. 2G). This suggested that the serines were not involved in the translocation of Myc-YAPS5A from cytoplasm to nucleus.

### Riluzole decreases phosphorylation of YAP at serine 127

YAP phosphorylation at serine 127 by Akt or Lats causes cytoplasmic retention and inhibits YAP from activating pro-apoptotic genes^34,38,39,49^. We show that Riluzole decreases the phosphorylation of YAP at serine 127 (Fig. 2I,J). The decrease in S127 phosphorylation thereby prevents cytoplasmic sequestration. Together the data in Fig. 2 demonstrates that Riluzole decreases S127 phosphorylation and affects the change in the localization of YAP to nucleus by decreasing cytoplasmic sequestration.

### Riluzole-induced apoptosis is blocked by c-Abl kinase inhibitors

**Figure 3:**
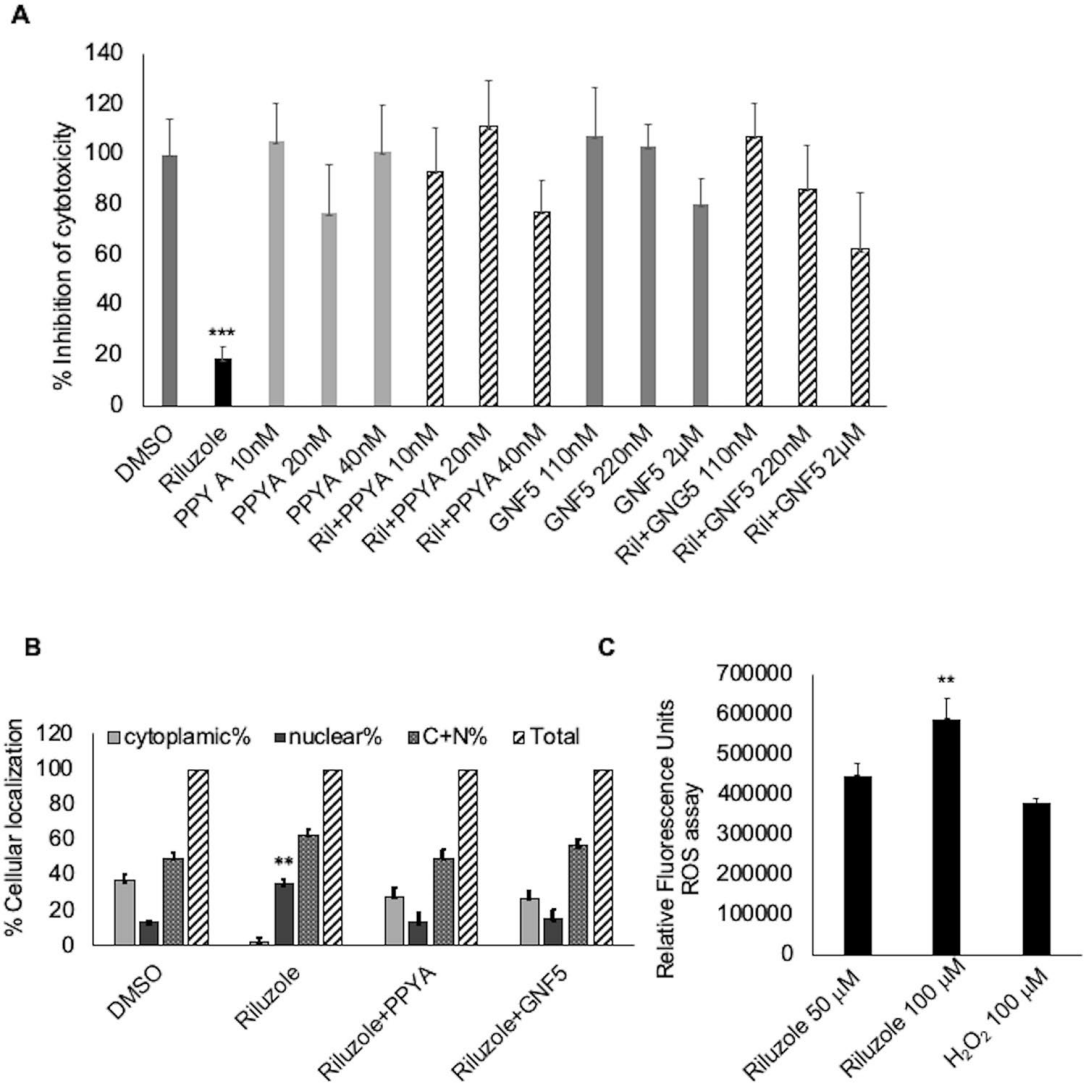
(**A**) Inhibitors of c-Abl Kinase block Riluzole-induced apoptosis. (**A**) Cytotoxicity was measured using MTT assay in LM7 cells in the presence of 100 μM Riluzole, or PPY A or GNF-5 or Riluzole + PPY A or Riluzole + GNF-5, p value < 0.0001***. (**B**) Subcellular localization of YAP in the presence of Riluzole (100 μM) or Riluzole + PPY A (20 nM) or Riluzole + GNF-5 (220 nM), p value < 0.05**. (**C**) Intracellular ROS was measured in LM7 cells treated with Riluzole or H_2_O_2_ as a positive control. These experiments were repeated three times with triplicate samples, p value < 0.05**.

YAP is shown to be phosphorylated by c-Abl kinase in response to DNA damage in HEK293 cells^50,51^. We wanted to test if the phosphorylation of YAP by c-Abl kinase plays a role in LM7 apoptosis, we performed a cell viability assay in the presence of various concentrations of two known c-Abl kinase inhibitors, PPY A and GNF-5. We tested both PPY A or GNF-5 alone or in the presence of Riluzole. The results, presented as percent of control sample, show that PPY A or GNF-5 did not significantly affect the viability of the cells on their own but prevented Riluzole-induced loss of viability suggesting that c-Abl kinase may be involved in Riluzole-induced apoptosis (Fig. 3A). We also measured the effect of c-Abl kinase inhibitors on Riluzole-induced nuclear localization of YAP. Both PPY A and GNF-5 were able to prevent Riluzole-induced nuclear localization of YAP (Fig. 3B) suggesting the involvement of c-Abl kinase in Riluzole induced apoptosis.

### Riluzole rapidly induces oxidative stress in LM7 cells

In hepatocellular carcinoma, Riluzole inhibited proliferation by elevating oxidative stress in vitro and in Huh7 xenograft model^18^. We found that inhibitors of c-Abl kinase blocked Riluzole-induced cytotoxicity. It is well known that c-Abl kinase is activated under conditions that induce DNA damage, such as an increase in oxidative stress^51^. We tested if Riluzole induces oxidative stress in osteosarcoma cells by measuring intracellular ROS. Riluzole induced significant increase in ROS at 50 μM, which was further enhanced at 100 μM (Fig. 3C). At 50 μM Riluzole induced comparable levels of ROS as 100 μM H_2_O_2_. This data clearly demonstrated that Riluzole increases oxidative stress, which may lead to the activation of c-Abl kinase.

### Riluzole-induced apoptosis is blocked by the knockdown of c-Abl Kinase

**Figure 4:**
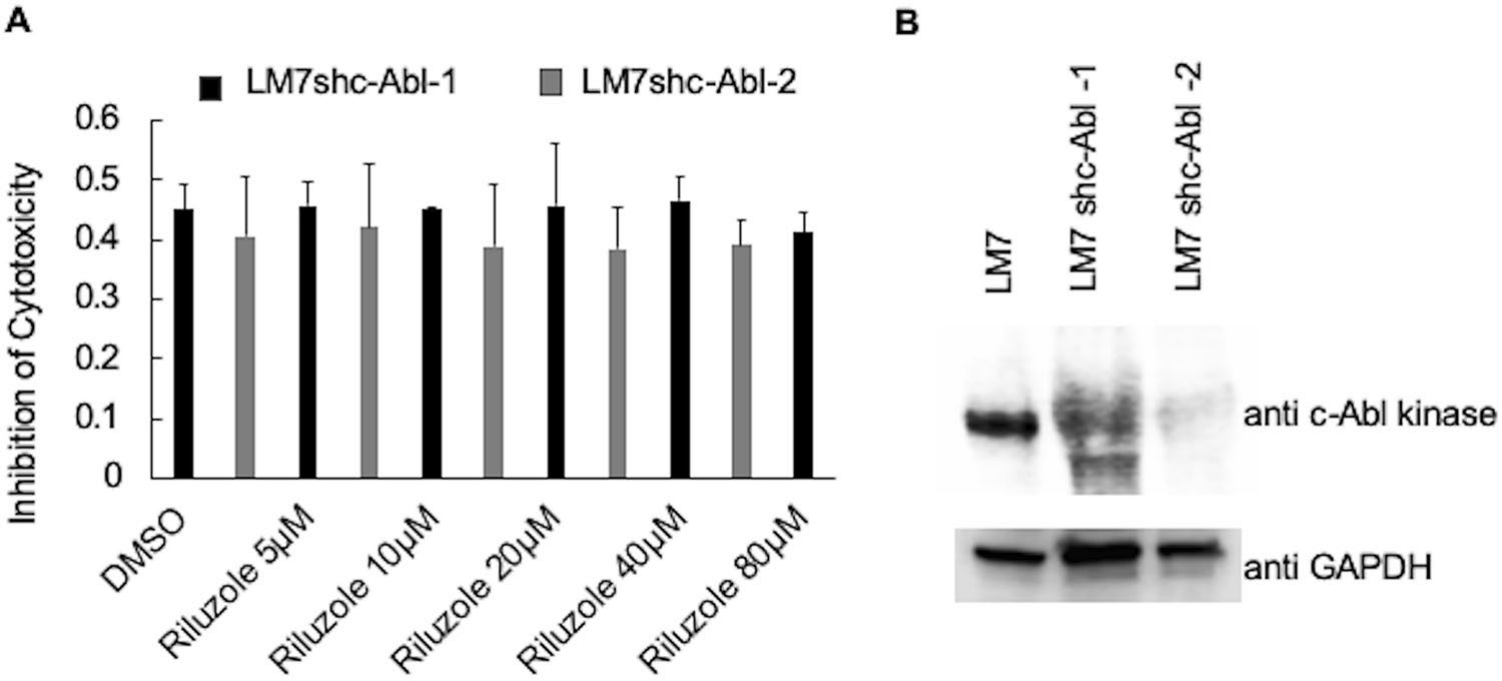
c-Abl Kinase is required for Riluzole-induced apoptosis in LM7 Cells. (**A**) LM7 cells with c-Abl kinase knockdown were tested for Riluzole sensitivity at various doses as indicated in a MTT assay. Data for two independent selected stable cells lines, LM7shc-Abl-1 and LM7shc-Abl-2, is shown. (**B**) Western blot of c-Abl kinase knockdown and GAPDH is shown as a control.

To further confirm the role of c-Abl kinase in Riluzole-induced apoptosis in LM7 cells, we knockdown c-Abl kinase by using shc-Abl kinase and selected stable LM7 cell lines by screening for the expression of c-Abl kinase. We selected two stable cell lines represented as LM7shc-Abl-1 and LM7shc-Abl-2, and tested the cell viability in the presence of various doses of Riluzole. The results show that Riluzole failed to induce apoptosis even at very high doses of 80 μM (Fig. 4A) for both cell lines, LM7shc-Abl-1 and LM7shc-Abl-2. The expression of c-Abl kinase protein in the WT cells and LM7shc-Abl kinase knockdown of two cell lines show significant reduction in c-Abl kinase protein levels with GAPDH as a loading control (Fig. 4B). The results of this experiment demonstrate that c-Abl kinase is necessary for Riluzole-induced apoptosis.

### Riluzole activates c-Abl kinase to phosphorylate YAP at tyrosine 357

**Figure 5:**
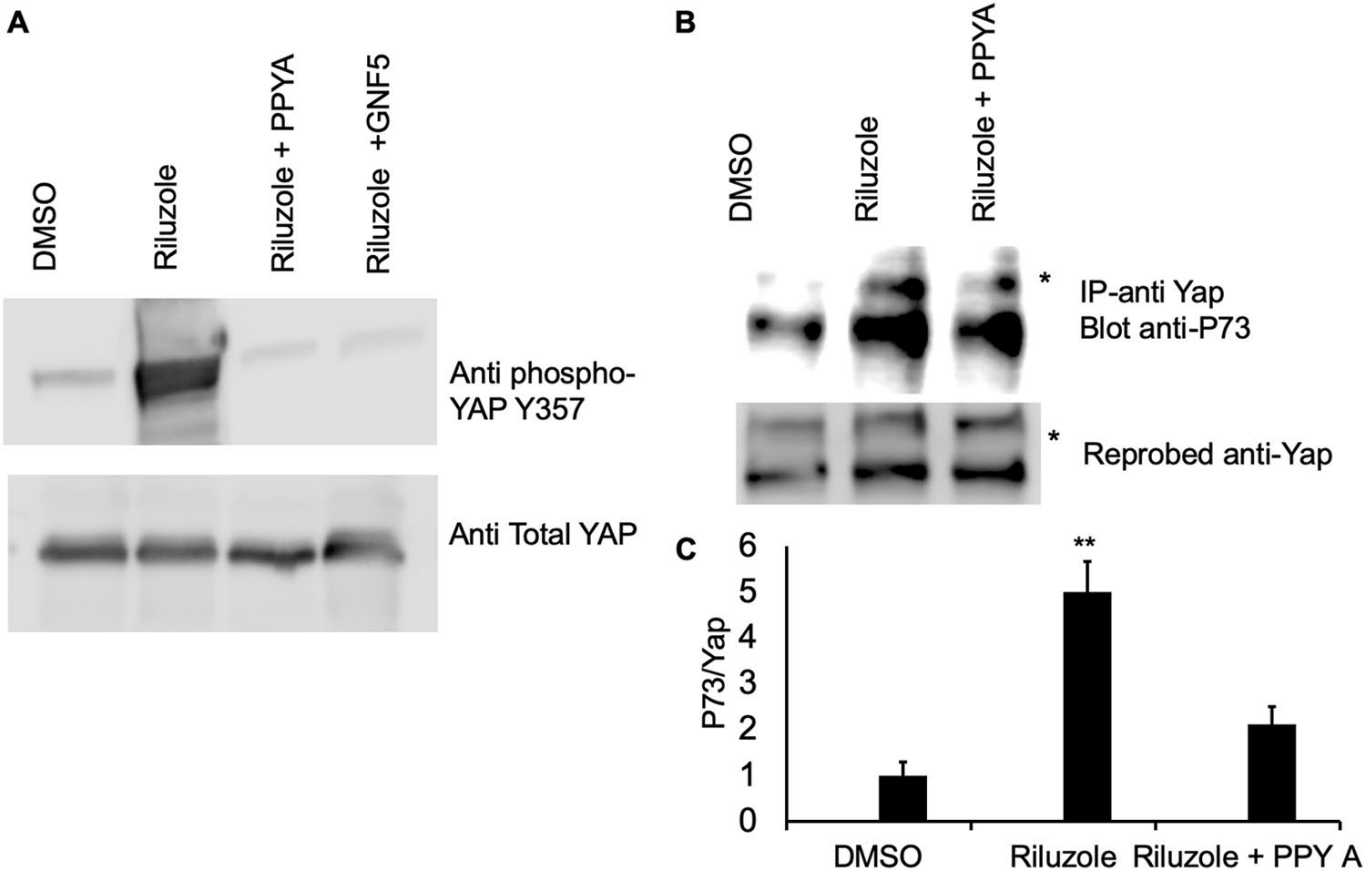
Riluzole induces phosphorylation of YAP at Y357 to promote binding of YAP with p73. (**A**) Whole cell extracts of treated LM7 cells with DMSO, Riluzole (50 μM) or Riluzole (50 μM) + PPY A (20 nM) were blotted with rabbit anti-YAP (phosphoY357) antibody and the blot was reprobed with mouse anti-YAP antibody. (**B**) Co-immunoprecipitation was performed to assess interaction between endogenous YAP and p73 using whole cell extracts. Samples were immunoprecipitated with mouse anti-YAP antibody and the western blot was probed with rabbit anti-p73 antibody, the blot was reprobed with mouse anti-YAP antibody. An asterisk next to the band indicates a nonspecific band. (**C**) The densities of the immunoprecipitated p73 and YAP were quantified to measure the ratio of p73/YAP. These experiments were repeated three times to confirm the results, p value < 0.05**, *IP* immunoprecipitation.

We then assessed the phosphorylation status of YAP at Y357 after Riluzole treatment. LM7 cells were either treated with Riluzole or the combination of Riluzole with either c-Abl kinase inhibitors or DMSO as a control. Whole cell extracts were prepared and immunoprecipitation was carried out using anti YAP antibody. The immunoprecipitated samples were analyzed by western blot using anti phopsho YAP Y357 antibody. The results of the IP and western blot clearly show a robust phosphorylation of YAP in Riluzole treated sample but not in control. Moreover, PPY A and GNF-5 blocked the Riluzole-induced phosphorylation of YAP at Y357 (Fig. 5A). This result clearly demonstrated that Riluzole activates c-Abl kinase, which phosphorylates YAP at Y357.

### Riluzole promotes YAP interaction with p73

We further tested if Riluzole promoted the interaction between YAP and P73 in a co-immunoprecipitation assay. Extracts made from LM7 cells treated with either DMSO as control, Riluzole or Riluzole + PPY A were co-immunoprecipitated using anti-YAP antibody. The immunoprecipitated samples were analyzed using a western blot with anti-p73 antibody. The top panel in Fig. 5B shows an increased p73 with Riluzole treatment which is partially blocked by PPY A in Riluzole + PPY A. The lower panel shows the same blot reprobed with anti-YAP antibody to assess the amount of immunoprecipitated YAP (Fig. 5B).The ratio of densities of p73 to YAP shows the highest interaction between YAP and p73 in the presence of Riluzole, and a decrease in the interaction between YAP and p73 is seen in the presence of PPY A (Fig. 5C). This data demonstrates that Riluzole promotes interaction between YAP and p73 as an early molecular event to induce apoptosis in osteosarcoma (model Fig. 7).

### Riluzole induces Bax expression

**Figure 6:**
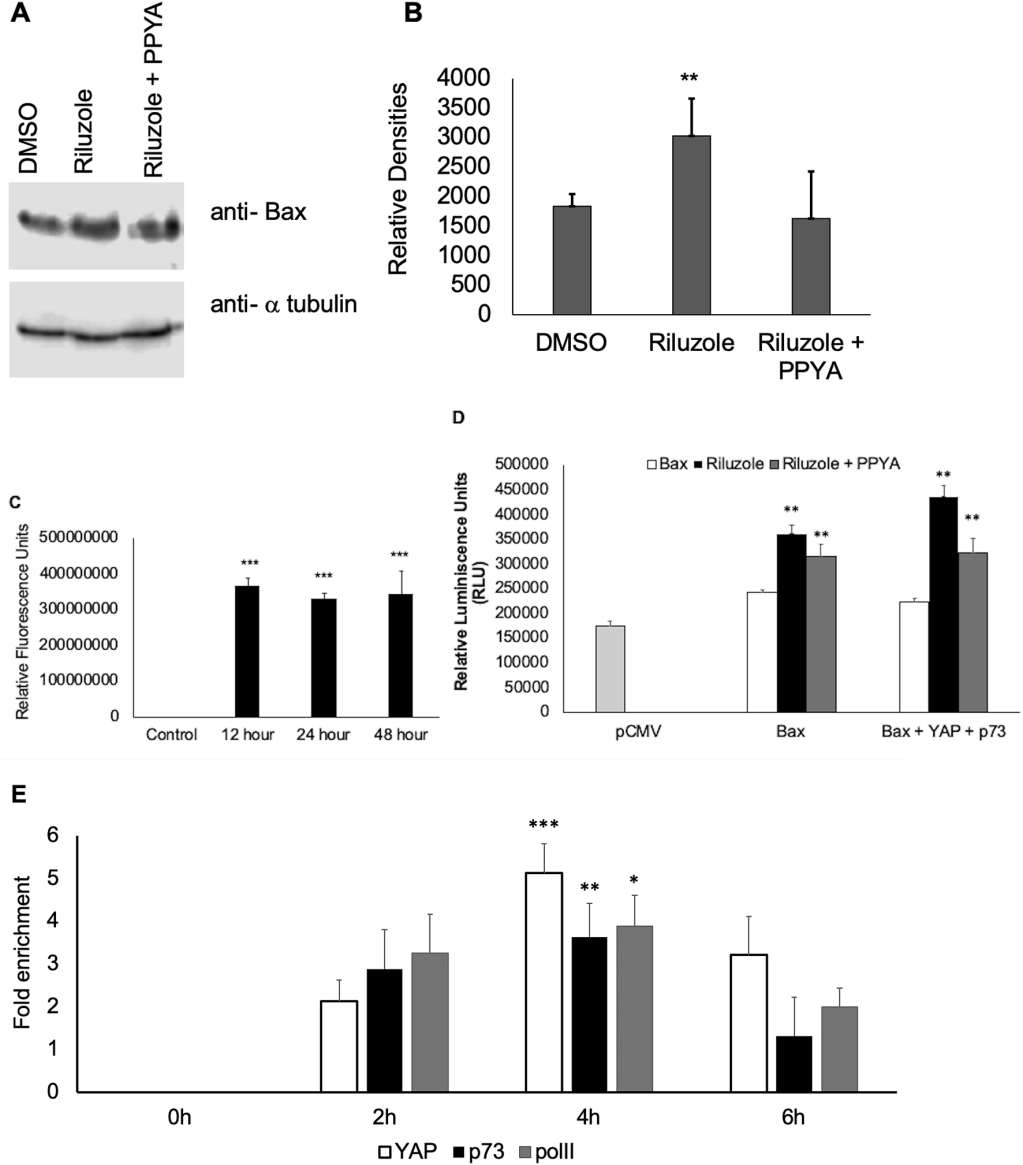
Riluzole enhances Bax expression by inducing Bax promoter activity in LM7 cells. (**A**) Whole cell extracts from LM7 cells were probed with anti-Bax antibody to determine the effect of Riluzole (50 μM) and Riluzole (50 μM) + PPY A (20 nM) on Bax expression, α tubulin is used as a loading control. (**B**) Bar graph showing densities of bands from 3 independent experiments. (**C**) Caspase assay represents data from 3 independent experiments, p value = 0.003***. (**D**) Bax promoter driven luciferase expression was measured in LM7 cells. LM7 cells were treated with DMSO or Riluzole (50 μM) or Riluzole (50 μM) + PPY A (20 nM) for 24 h, p value < 0.05**. (**E**) Fold enhancement of the Bax promoter activity was measured using Chromatin Immunoprecipitation assay using anti YAP, anti p73 and or Polymerase II antibodies followed by qPCR to detect and quantify fold induction of Bax promoter. Representative data of three independent experiments is shown, p value < 0.5* p value < 0.05**. p value < 0.005***.

**Figure 7:**
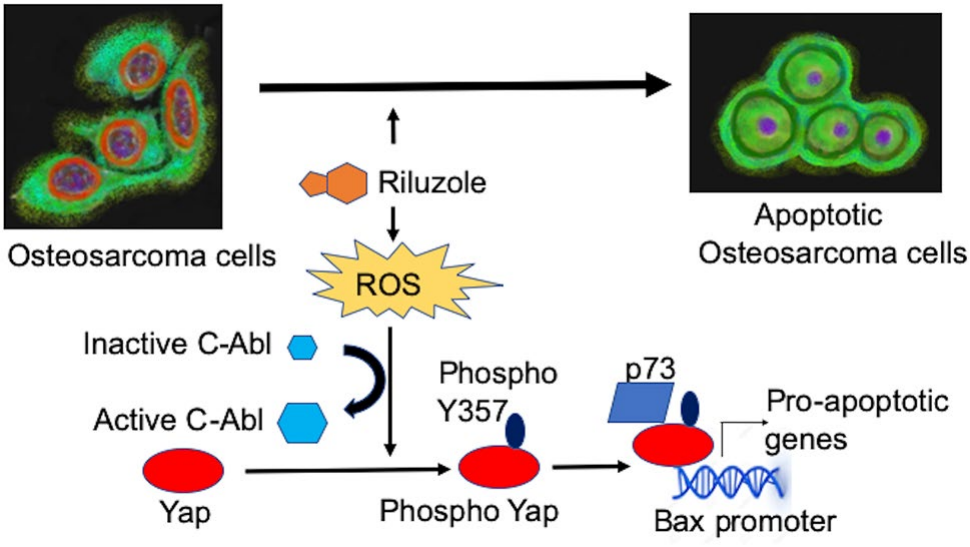
Mechanism of action of Riluzole-induced apoptosis in osteosarcoma. A schematic representation of the mechanism of Riluzole action is shown.

YAP overexpression is known to enhance p73 mediated Bax expression in breast and colon cancer cell lines upon exposure to DNA damaging agents^34,52^. Moreover, YAP phosphorylation at Y357 facilitates binding to p73 to activate transcription of pro-apoptotic genes such as Bax, DR5, and PUMA^45,53,54^. To determine the effect of Riluzole on Bax expression we performed western blots using anti-Bax antibody on LM7 whole cells extracts. Bax expression in LM7 cells was enhanced by Riluzole and was partially blocked by c-Abl kinase inhibitor, PPY A (Fig. 6A,B). As expected, increase in caspase activation was also observed with increased duration of Riluzole treatment at 12 h, 24 and 48 h in LM7 cells (Fig. 6C). In a luciferase reporter assay, Riluzole increased Bax promoter driven luciferase expression, however, luciferase expression was significantly enhanced by Riluzole when YAP and p73 were exogenously overexpressed in LM7 cells. The increase in Bax luciferase expression with Riluzole in LM7 cells expressing exogenous YAP and p73 was blocked by PPY A suggesting the involvement of c-Abl kinase in the activation of Bax promoter through phosphorylation of YAP at Y357 (Fig. 6D). To assess the transcriptional regulation at the endogenous Bax promoter by YAP, p73 and RNA polymerase II activity upon Riluzole treatment, a chromatin immunoprecipitation (ChIP) assay was performed followed by qPCR to detect and quantitate Bax promoter. Compared to the untreated samples, 2–threefold enhancements in the occupancy of the Bax promoter by YAP, p73 and RNA polymerase II were observed at 2 h, 3.6–5-fold enhancements were observed at 4 h and then a decrease to 1.3–3.2-fold enhancements were observed at 6 h after Riluzole treatment. The maximum occupancy by YAP, p73 and RNA Polymerase II was observed at 4 h after Riluzole treatment (Fig. 6E). The data in Fig. 6 demonstrated that Riluzole induced Bax expression by promoting interaction between YAP and p73 which bind to the Bax promoter along with RNA polymerase II to activate transcription of Bax gene. A model representing the mechanism of action of Riluzole-induced apoptosis in osteosarcoma is shown in Fig. 7 (“Supplementary information”).

## Discussion

Our study demonstrates the mechanism of action of Riluzole-induced apoptosis in osteosarcoma, which involves phosphorylation of YAP by c-Abl kinase, to promote interaction with p73 leading to the activation of pro-apoptotic genes. Our data showed that Riluzole changed the localization of YAP from cytoplasmic to nuclear by phosphorylating YAP at tyrosine 357. Riluzole does this by activating c-Abl kinase, which is known to be induced by stress under DNA damage conditions. Phosphorylation of serine 127 is known to sequester YAP in cytoplasm and phosphorylation at serine 381 is shown to promote ubiquitin mediated degradation of YAP^34,38,55^. The 5SYAP mutant used in our localization studies included mutations of serine at 127 and 397 demonstrating that these residues are not important for YAP localization to nucleus, prompting the involvement of phosphorylation of residues other than the 5 mutated serine residues (serine 61, 109, 127, 164, and 397). Based on our data, we believe that nuclear localization of YAP was facilitated by changes in phosphorylation, both by a decrease in S127 phosphorylation and an increase in Y357 phosphorylation. This effectively promoted association of YAP with p73 to activate pro-apoptotic genes.

Earlier studies by others demonstrated YAP phosphorylation by c-Abl kinase as a critical step in inducing apoptosis under DNA damage conditions. c-Abl kinase phosphorylated YAP at tyrosine 357 which stabilized YAP to promote its interaction with p73^51^. Our data shows an increase in ROS production upon Riluzole treatment, which may lead to DNA damage. Moreover, we showed the inhibition of Riluzole-induced apoptosis in the presence of two specific c-Abl kinase inhibitors, PPY A and GNF-5, demonstrating the involvement of c-Abl kinase in Riluzole-induced apoptosis. This finding was strengthened by data from c-Abl knockdown in LM7 cells in which Riluzole failed to induced apoptosis even at high doses, supporting the necessity of c-Abl kinase in Riluzole-induced apoptosis.

YAP was shown to associate with p73 through the interaction of the WW domain YAP and PPPY motif of P73^46^. Furthermore, YAP was shown to stabilize p73 by competing for binding to E3 ubiquitin ligase, Itch, to prevent p73 degradation^44^. Subsequently, in cisplatin-induced c-jun-mediated apoptosis, YAP was shown to play a critical role by stabilizing p73^56^. Interestingly, a switch in YAP activity from anti-apoptotic to pro-apoptotic was shown to involve phosphorylation of YAP by c-Abl kinase under conditions of DNA-damage. Moreover, YAP was shown to recruit p73 to promoters to activate the transcription of apoptotic genes and also promoted p300-mediated p73 acetylation to enhance the transcriptional potential of p73^52^. YAP phosphorylated at Y357 binds to P73 to activate transcription of pro-apoptotic genes such as Bax, DR5, and PUMA^45,53,54,57^. Therefore, YAP regulates stability of p73, enhances transcription activity of p73 by acetylation and binds to pro-apoptotic genes promoters with p73 to activate transcription of pro-apoptotic genes^45,52^. Consistent with these findings, our data using chromatin immunoprecipitation assay followed by qPCR, has demonstrated that Riluzole increased occupancy of endogenous Bax promoter by YAP and p73, which recruited RNA polymerase II to activate transcription from the Bax promoter.

Although Riluzole induces apoptosis in osteosarcoma in the xenograft mouse model, it needs to be determined if Riluzole activates c-Abl kinase and phosphorylates YAP at Y357 to induce apoptosis in vivo^58^*.* Riluzole has been shown to induce apoptosis through ER stress in prostate cancer cell lines^15^. In hepatocellular carcinoma, Riluzole increased reactive oxygen species (ROS)^18^. It would be interesting to investigate if Riluzole activates c-Abl kinase in prostate cancer, hepatocellular carcinoma or in melanoma during apoptosis. Interestingly, Riluzole has been shown to cause an increase in cytosolic Ca^2+^ in human osteosarcoma MG63 cells^59^. It is conceivable that increase in cytosolic Ca^2+^ may be a very early event in the action of Riluzole. Our data demonstrates that Riluzole increases oxidative stress after 2 h of treatment with Riluzole in osteosarcoma, unlike the 24 h of treatment in hepatocellular carcinoma^18^. This data is also supported by the detection of YAP phosphorylation at Y357 after 2 h of Riluzole treatment. Furthermore, in prostate cancer cells, Riluzole has been shown to cause caspase activation via endoplasmic reticulum (ER) stress^15^. More recently, Riluzole is shown to have anti-tumorigenic effects in prostate cancer cells via ER stress-induced degradation of androgen receptor, thereby decreasing expressions of target genes such as prostate specific antigen (PSA)^60^. Our findings have demonstrated that Riluzole action on osteosarcoma cells is 2-fold: (1) deprives cells of glutamate so the growth signaling is inhibited^8^, (2) induces apoptosis by activating c-Abl kinase to phosphorylate YAP at Y357 for the regulation of pro-apoptotic gene transcription. Our studies provide a mechanism of action of Riluzole in inducing apoptosis in osteosarcoma cells. We believe that the use of Riluzole in combination with surgery may show a favorable outcome for therapy for osteosarcoma.

## Materials and methods

### Cell culture

LM7 cells^9^ were maintained in DMEM supplemented with 4.5% glucose, 1 mM pyruvate, 10% fetal bovine serum, 2 mmol/l GlutaMAX-I, 100 units/ml penicillin and 100 μg/ml streptomycin^9^. Cells were passaged every 4 days. Cells were maintained at 37 °C with 95% air and 5% CO_2_. The Saos-2-LM7 cells were provided by Dr. Eugenie Kleinerman, MD Anderson Cancer Center, Houston.

### Drug treatments

For TUNEL assay or MTT assay the cells were seeded and 24 h later the cells were treated with drugs such as 100 μM Riluzole, or PPY A (10 nM, 20 nM or 40 nM) or GNF-5 (110 nM, 220 nM 0r 2 μM) as specified in each experiment for 24 h. For western blots and co-immunoprecipitation assays, the cells were seeded and after 24 h the cells were treated with 100 μM Riluzole or 20 μM PPY A or 220 nM GNF-5 for 2 h. When combination of c-Abl kinase inhibitors were used with Riluzole, the inhibitors were added 15 min prior to the treatment of Riluzole. All drugs were purchased from R&D systems, Minneapolis, MN.

### TUNEL assay

The TUNEL assay was performed using the in situ cell death detection kit from Roche as per manufacturer’s instructions. Briefly, 1.5 × 10^4^ cells seeded onto polylysine coated coverslips in 24 well plates, after 24 h were treated with 100 μM Riluzole for 24 h. The cells were fixed with 4% paraformaldehyde and permeabilized in 0.2% Triton X-100, the fixed cells were incubated with the TUNEL reagent containing TMR red labeled nucleotides at 37 °C for 1 h. The samples were washed in 1× PBS three times and mounted in mounting media containing DAPI. Fluorescent images were captured using a Zeiss fluorescence microscope at 20× magnification. The total number of DAPI positive cells and total number of TUNEL positive cells were counted from at least five images from each sample. Each experiment was repeated four times with triplicate samples.

### Subcellular localization

Retroviruses for Myc-tagged WT YAP (Addgene # 33091) and 5SA YAP (Addgene # 33093) in pQCXIH vector (from the laboratory of Dr. Kung-Lian Guan) were produced in 293-GP cells and pseudotyped with VSV-G. LM7 cells were infected with different titers of Myc-tagged WT YAP or Myc-tagged 5SA YAP retrovirus and stable cells were selected in media containing 500 μg/ml hygromycin. Subcellular localization of endogenous YAP or stably expressed Myc-tagged WT-YAP or Myc-tagged YAPS5A was measured in LM7 cells grown in 0.5% serum or in 10% serum. The cells were treated with DMSO or Riluzole (50 μM) for 1 h, fixed and stained with either anti YAP (A and B) or anti-C Myc monoclonal antibody (9MA1-980, Thermo Fisher Scientific) and secondary antibodies Alexa 488 (A-11001, Thermo Fisher Scientific). Fluorescence microscopy was performed on blinded samples, images were captured at 40X, and YAP localization was analyzed. The cells were scored for either cytoplasmic or nuclear or both cytoplasmic and nuclear (C + N) localization. We used DAPI to count the total number of cells.

### Knockdown of YAP and c-Abl kinase, shRNA

YAP 1 expression was knocked down using Lentivirus vector TRC version 2, pLKO.1-puro vector, expressing shRNA for YAP, non-target shRNA (SCH002) was used as control. The lentiviruses for YAP 1 and control were produced using 293 T cells using pVSV-G, pLP1 and pLP2 as helper plasmids. LM7 cells were infected with various titers of viruses expressing either shRNA for YAP 1 or virus containing control shRNA. Stable cells were selected in media containing puromycin (1.5 μg/ml). Stable cells containing the control shRNA or expressing shRNA for YAP were isolated as individual colonies and tested for expression of YAP. ABL1 MISSION shRNA Lentiviral Transduction Particles c-Abl oncogene 1, non-receptor tyrosine kinase, (Clone ID:NM_005157.3-3354s1c1 (TRCN0000288647 CCGGCCTCAGTTCGGTGAAGGAAATCTCGAGATTTCCTTCACCGAACTGAGGTTTTTG) in pLKO.1 vector) were purchased from Millipore Sigma. Viral particles with c-Abl shRNA or control shRNA were titrated to infect LM7 cells. Stable cells were selected in media containing puromycin (1.5 μg/ml). Twenty five colonies expressing shRNA for c-Abl kinase were selected and four colonies were expanded and tested for expression of c-Abl kinase by western blotting using mouse anti c-Abl kinase (MAB1130, Millipore Sigma).

### MTT assay for measuring cytotoxicity

LM7 cells (300 cells/well) seeded in 96 wells in plates. After 24 h the cells were treated with either DMSO, 100 μM Riluzole, PPY A (10 nM, 20 nM or 40 nM) or GNF-5 (110 nM, 220 nM 0r 2 μM), 100 μM Riluzole + PPY A (10 nM, 20 nM or 40 nM) or 100 μM Riluzole + GNF-5 (110 nM, 220 nM or 2 μM) indicated concentrations in fresh media. The cells were incubated for 48 h and MTT assay was performed. 10 μl of MTT (3-(4,5-dimethylthiazol-2-yl)-2,5-diphenyltetrazolium bromide) solution was added to cells at 5 mg/ml concentration. The cells were incubated for 2 h and the precipitated crystals were solubilized in DMSO and the absorbance was measured at 570 nm with a reference wavelength at 670 nm.

### Reactive Oxygen Species (ROS) assay

LM7 cells were seeded in a 24-well plate (120,000/well). After 24 h, the cells were either not treated for control or treated with Riluzole 50 μM or 100 μM for 2 h. Reactive Oxygen Species were measured by OxiSelect Intracellular ROS assay Kit (Green Fluorescence) (Cell Biolabs, Inc). Briefly, the cells were washed with 1× PBS and loaded with cell permeable fluorogenic probe 2′7′-dichlorodihydrofluorescin diacetate (DCFH-DA) in serum-free media and incubated for 1 h. 100 μM H_2_O_2_ was added during the incubation to treat cells for 30 min. The cells were washed and lysed in 1× lysis buffer diluted in serum-free media and the fluorescence was measured using fluorescence plate reader at 480 nm/530 nm using Molecular device Spectramax i3 plate reader. Background fluorescence from cells was subtracted from the Riluzole and H_2_O_2_ treated samples. The experiment was repeated three times with triplicate samples.

### Western blotting

Western blotting was carried out using whole cell extracts made in buffer (400 mM KCl, 10 mM Tris–HCl [pH 7.9], 5% glycerol, 0.25% NP-40, 0.2 mM EDTA, 0.5 mM phenylmethylsulphonyl fluoride, 0.2 mM sodium vanadate, 50 μM sodium fluoride). Antibodies used were mouse anti-YAP antibody (ab56701, Abcam), rabbit anti-YAP (phospho Y357) antibody (ab62751, Abcam), rabbit anti-p73 antibody (ab14430, Abcam), mouse anti c-Abl kinase (MAB1130, Millipore Sigma), and rabbit anti-GAPDH (10494-1-AP, Proteintech). Anti-Bax antibody (50599-2-IG, Proteintech) Signal was detected using chemiluminescence using Pierce reagents. Western blots were read using C-Digit and bands were quantified by measuring the relative densities of the bands using Li-Cor software. The ratio of the phosphorylated protein to total protein was calculated and analyzed by Student’s T test.

### Co-immunoprecipitation and Western blotting

LM7 cells were grown in 10 cm dishes to 70–80% confluency and treated with either DMSO, Riluzole, PPY A, GNF-5, Riluzole + PPY A or Riluzole + GNF-5 for 2 h. Whole cell extracts were made in buffer (400 mM KCl, 10 mM Tris–HCl [pH 7.9], 5% glycerol, 0.25% NP-40, 0.2 mM EDTA, 0.5 mM phenylmethylsulphonyl fluoride, 0.2 mM sodium vanadate, 50 μM sodium fluoride) and diluted with a buffer without salt to bring the KCl concentration to 200 mM. Protein A beads pre-incubated with anti-YAP antibody were added to whole cell protein extracts with protease and phosphatase inhibitors were incubated for 2 h at 4 °C. The beads were washed with buffer (200 mM KCl, 10 mM Tris–HCl [pH 7.9], 5% glycerol, 0.25% NP-40, 0.2 mM EDTA, 0.5 mM phenylmethylsulphonyl fluoride, 0.2 mM sodium vanadate, 50 μM sodium fluoride) and immunoprecipitated sample was analyzed by western blot using either anti-phosphoYAPY357 antibody or anti-P73 antibody. The blots were reprobed with anti YAP antibody to quantify the amount of immunoprecipitated YAP protein (ab56701, Abcam).

### Caspase assay

LM7 cells were seeded at a density of 0.4 million per 10 cm dish and were treated next day with 100 μM Riluzole for 12 h, 24 h or 48 h. Caspase assay was performed using the Caspase-10 Fluorometric Assay Kit (BioVision K-124) as per the instructions of the kit. The caspase activity was normalized using protein concentration of the samples and the control values were subtracted from treated sample values.

### Luciferase reporter assay

LM7 cells were seeded in 24-well plate (60,000 cells/well), the following day 500 ng of luciferase reporter plasmid vectors, pCMV luciferase, or PGL3Bax luciferase along with 10 ng of TK Renilla luciferase plasmid to normalize for transfection efficiency or in combination with expression vectors. In LM7 cells, pBABEWTYAP, PBABEHAP73, and PGL3Bax were transfected using Lipofectamine 3000 reagents for 24 h. pGL3Bax luciferase transfected samples were treated with Riluzole (50 μM), Riluzole + PPYA (20 nM) or DMSO for control for 24 h. Cell lysates were prepared and assayed for luciferase according to the PIERCE Glow Firefly/Renilla luciferase assay and the plate was read at a 530 nm emission filter using Molecular device Spectramax i3 plate reader.

### Chromatin immunoprecipitation assay

LM7 cells grown to confluency (10 million cells) on 15 cm dishes were treated with 100 μM Riluzole for 2, 4 and 6 h. The cells were fixed using 1% formaldehyde then quenched with 125 mM glycine. The cells were scraped off in ice cold PBS and pelleted down. The pellet was resuspended in swelling buffer and dounced 20 times. The pelleted nuclei were resuspended in ChIP lysis buffer and sonicated for 40 pulses at 80% amplitude on dry ice. The sonicated samples were then diluted in RIPA buffer and input was withdrawn before processing the samples further. The samples were subjected to immunoprecipitation overnight at 4 °C using sepharose A/G beads with antibodies against YAP (Cell Signaling 49125), p73 (Abcam ab215038) or RNA polymerase II (Cell Signaling 26293). The samples incubated with beads alone were used as control reference sample. The immunoprecipitated samples were then subjected to RNA digestion overnight and purified using PCR purification kit (Qiagen). The purified samples were diluted and then used for qPCR with SYBR-green mix reagent. 2^−ΔΔ*CT*^ method was used to calculate fold differences between samples using beads alone as control. Bax promoter primers used were: Bax R: 5′-AGCTGCCACTCGGAAAAAGA-3′ and Bax F: 5′-AGGATGCGTCCACCAAGAAG-3′.

### Statistical analysis

The overall significance was determined by one-way ANOVA using SPSS software and significance between each group was determined by post-hoc analysis using Tukey’s test.

## Supplementary Information

Supplementary Information.
