# Supplementary material for: Riluzole-induced apoptosis in osteosarcoma is mediated through Yes-associated protein upon phosphorylation by c-Abl Kinase
Source: Sci Rep. 2021 Oct 25;11:20974. doi: 10.1038/s41598-021-00439-8 (PMC8546089; doi:10.1038/s41598-021-00439-8)

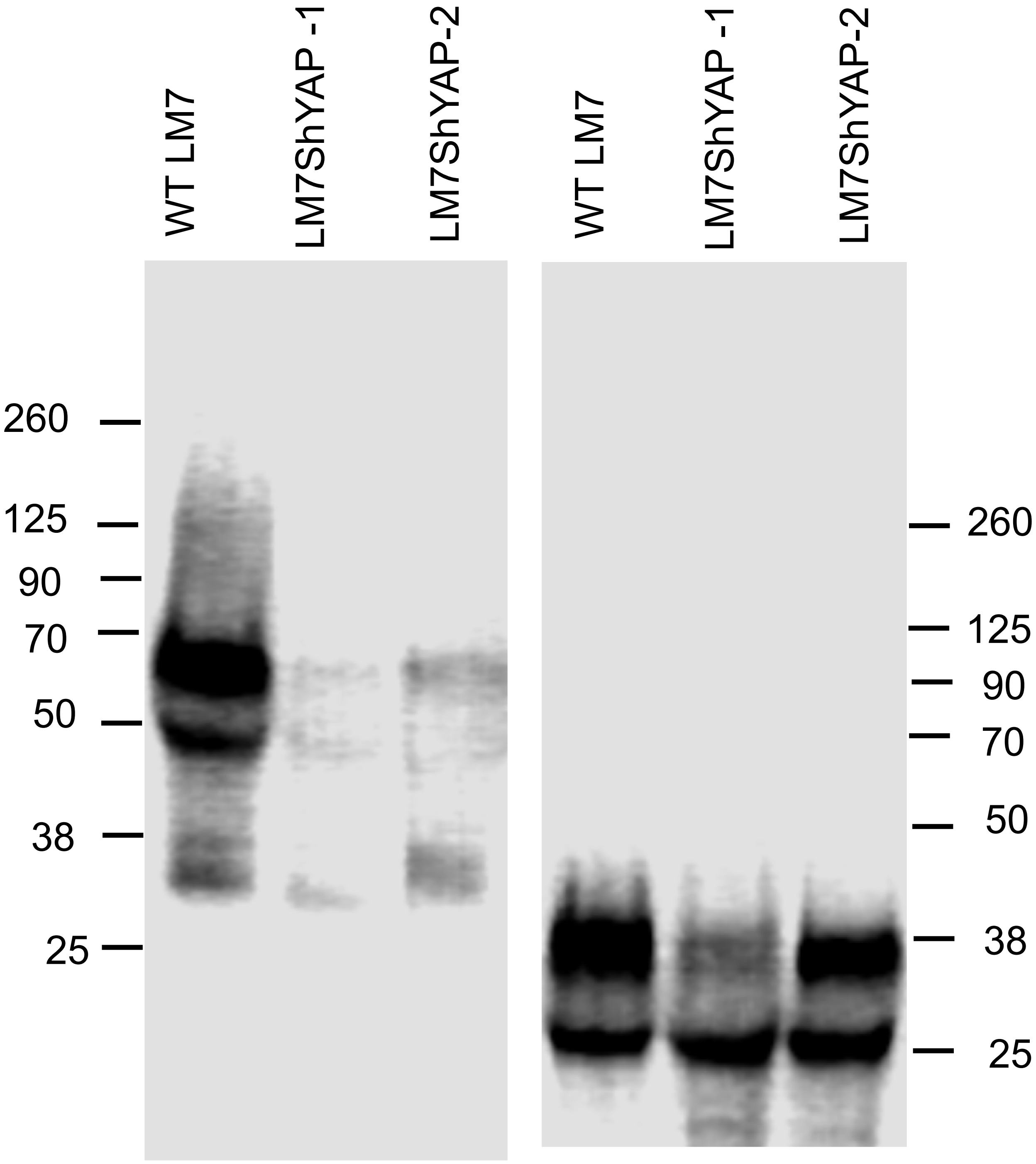
WT LM7
LM7ShYAP -1
LM7ShYAP-2
WT LM7
LM7ShYAP -1
LM7ShYAP-2
260
125
90
70
50
38
25
260
125
90
70
50
38
25

Figure 1D

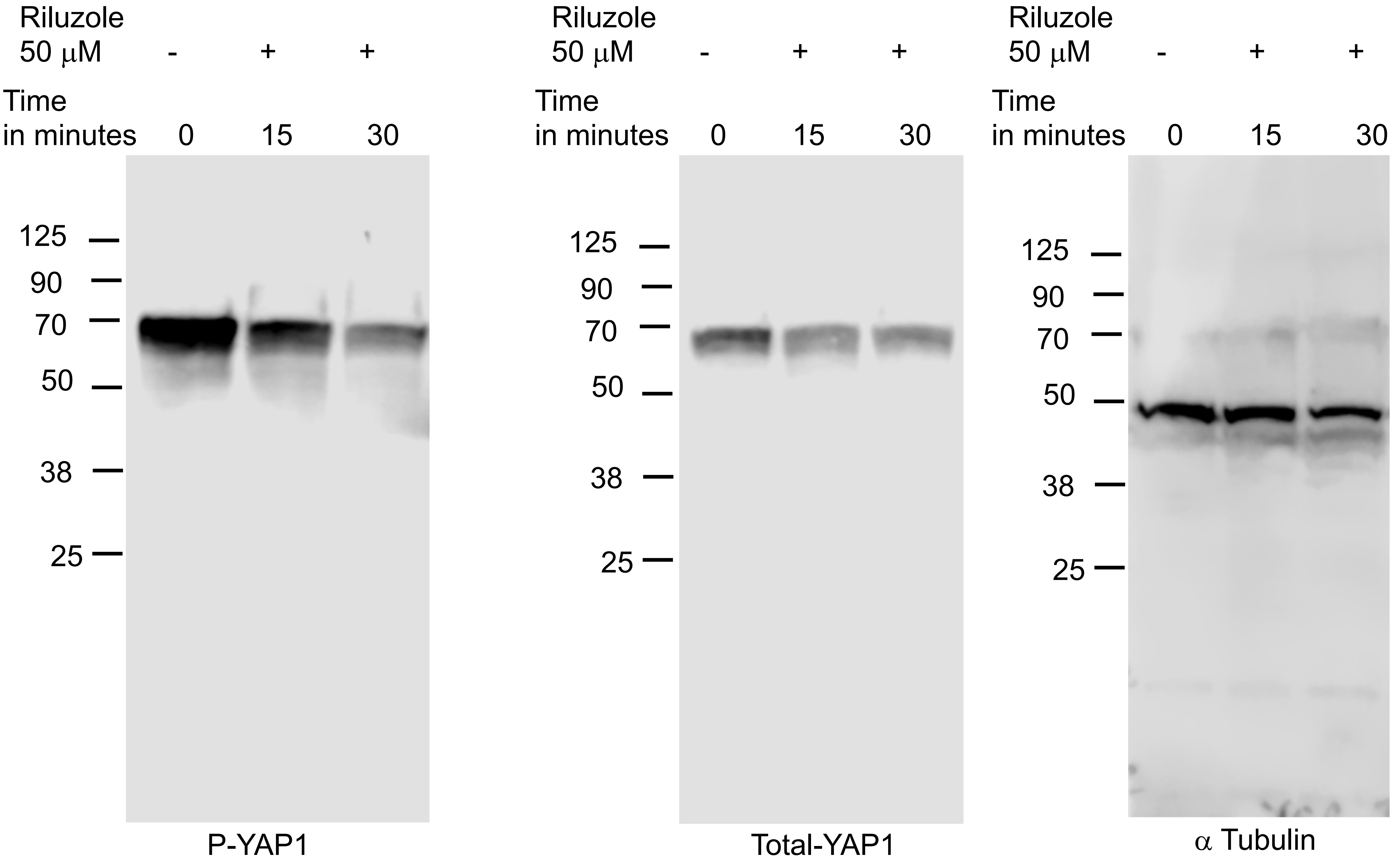
Riluzole
50 μM - + +
Time
in minutes 0 15 30
125
90
70
50
38
25
P-YAP1
Riluzole
50 μM - + +
Time
in minutes 0 15 30
125
90
70
50
38
25
Total-YAP1
Riluzole
50 μM - + +
Time
in minutes 0 15 30
125
90
70
50
38
25
α Tubulin

Figure 2I

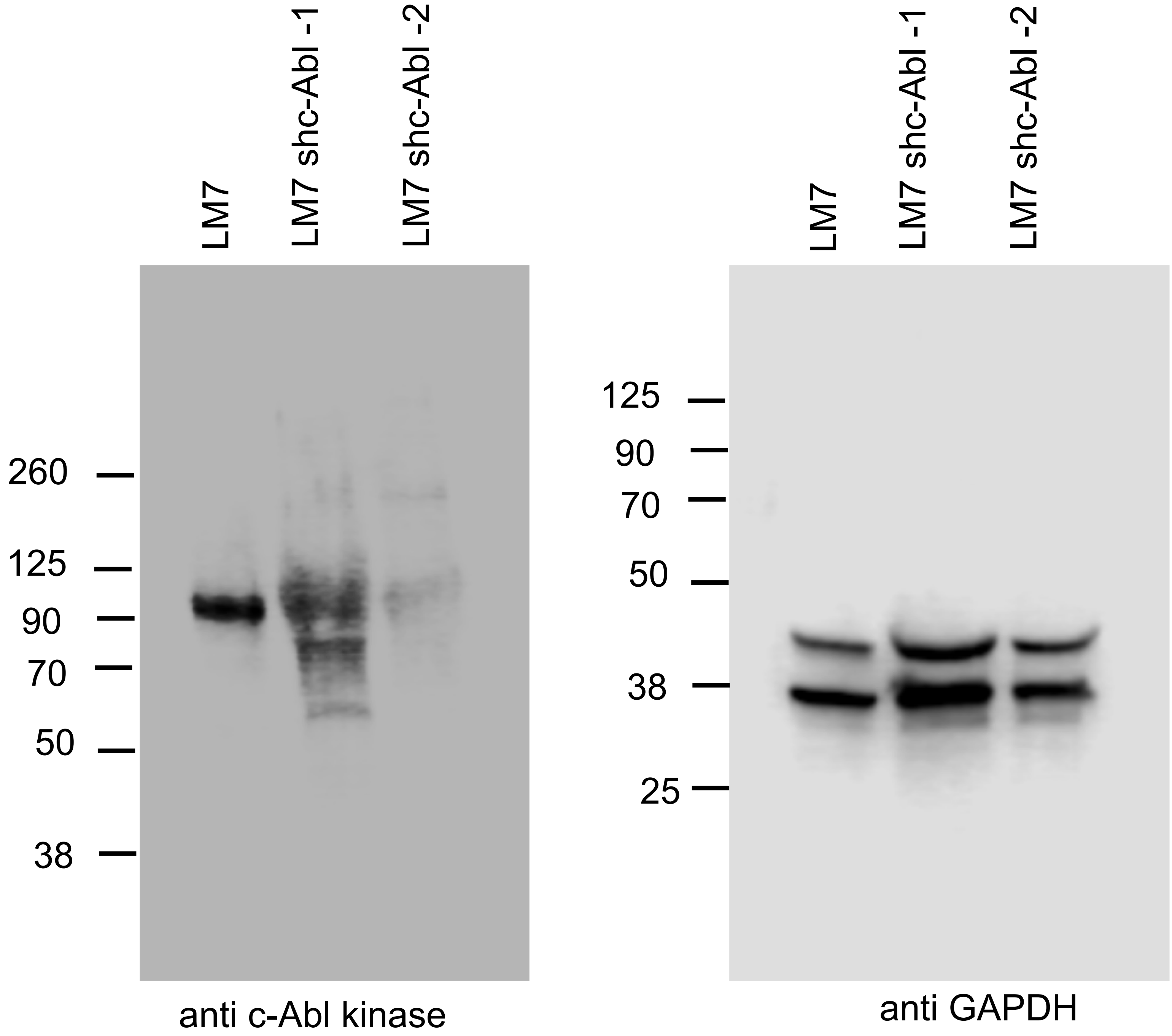

LM7
LM7 shc-Abl -1
LM7 shc-Abl -2
260
125
90
70
50
38
anti c-Abl kinase
LM7
LM7 shc-Abl -1
LM7 shc-Abl -2
125
90
70
50
38
25
anti GAPDH

Figure 4B

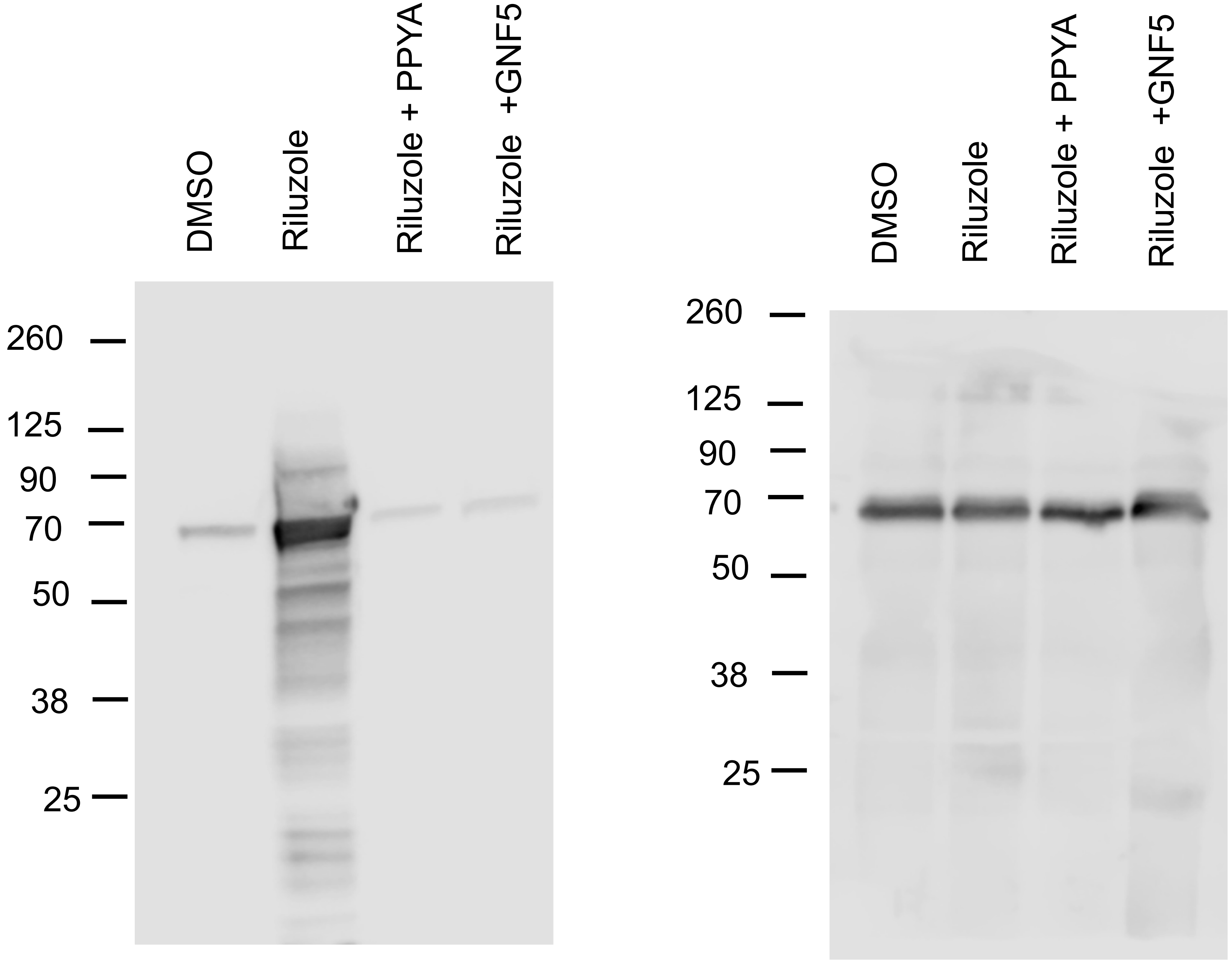

anti phospho-YAP Y357

Anti-YAP

Figure 5A

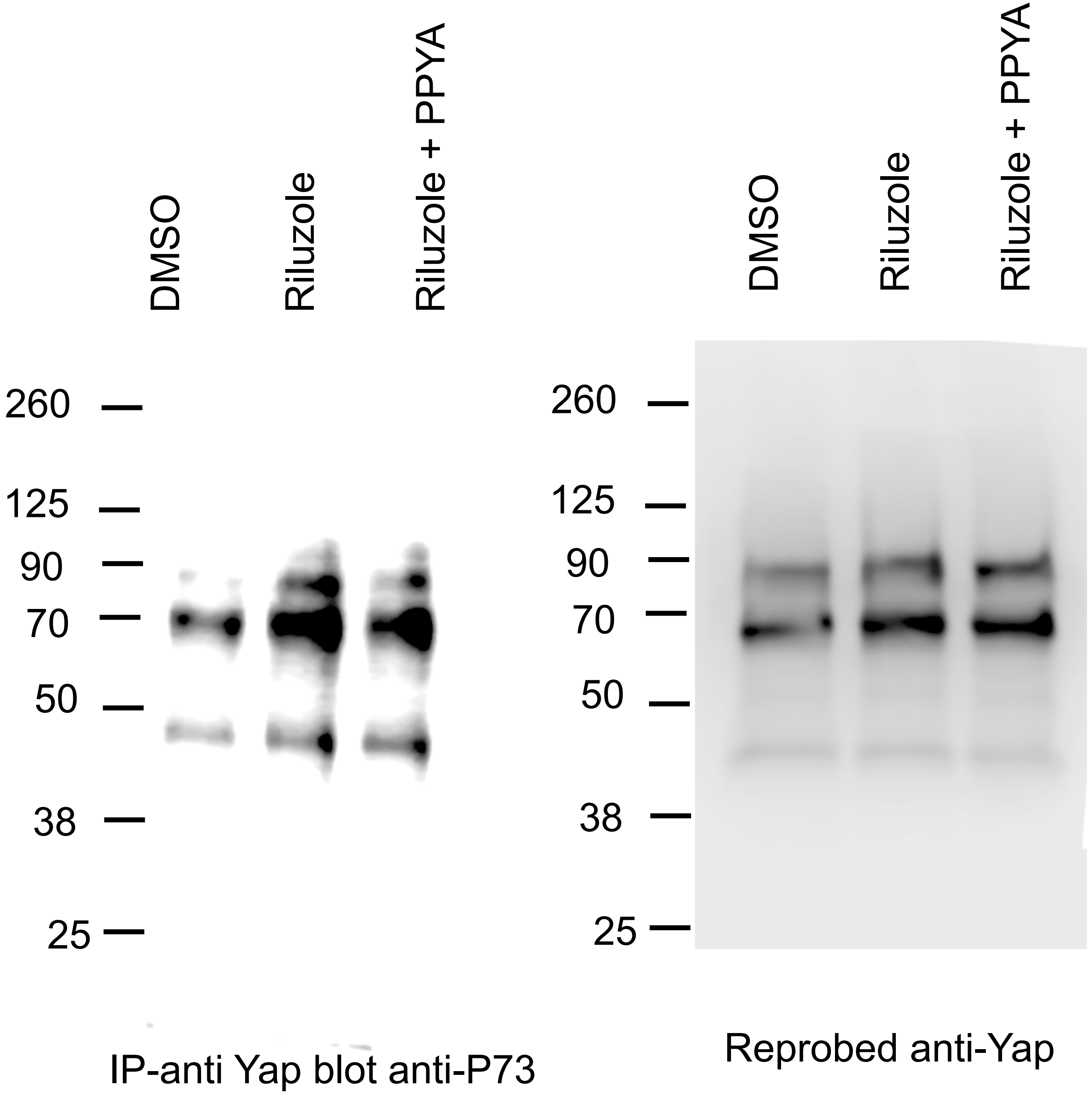
DMSO
Riluzole
Riluzole + PPYA
260
125
90
70
50
38
25
IP-anti Yap blot anti-P73
DMSO
Riluzole
Riluzole + PPYA
260
125
90
70
50
38
25
Reprobed anti-Yap

Figure 5B

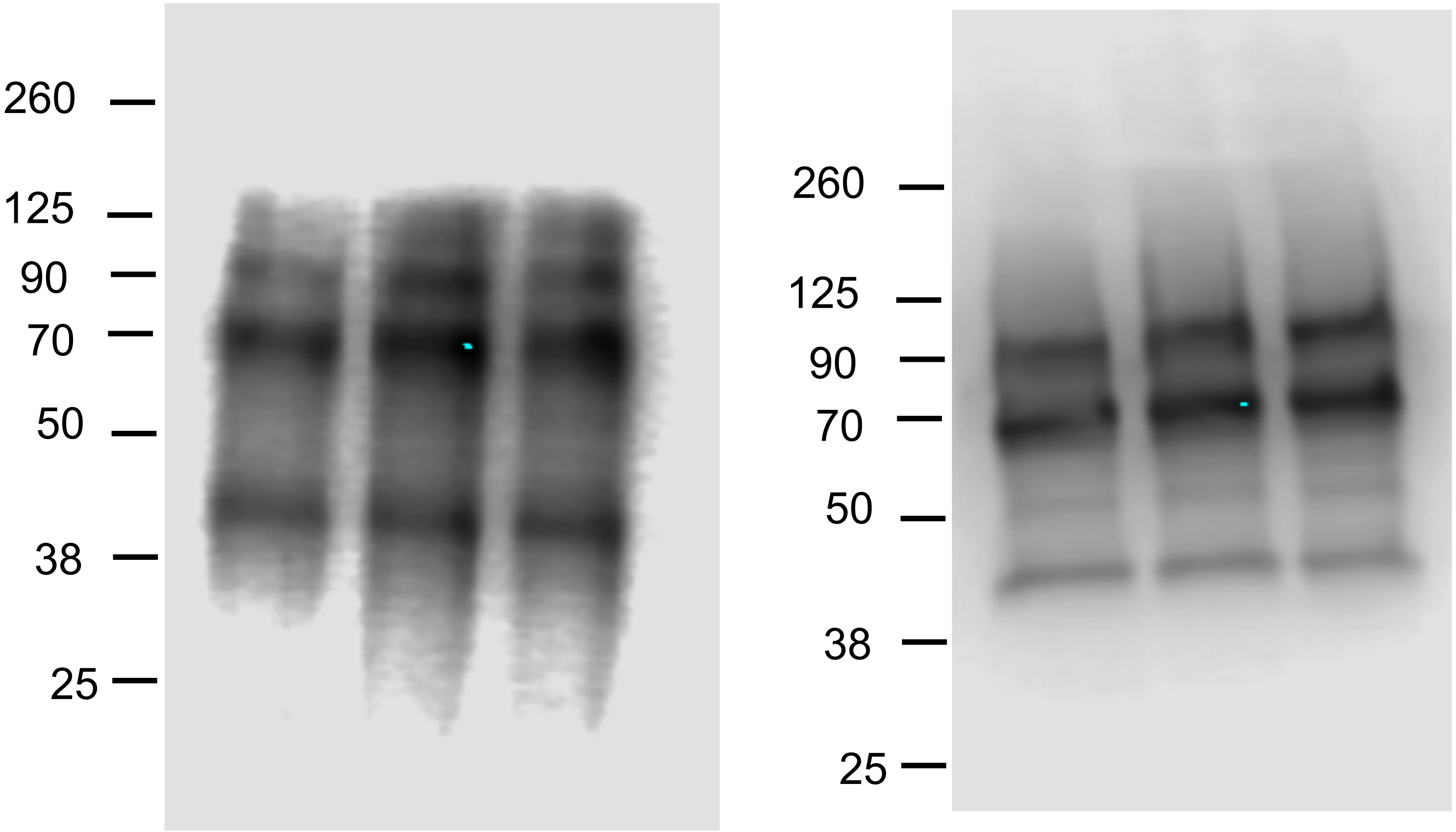

IP-anti Yap blot anti-P73

Reprobed anti-Yap

Long exposures Figure 5B

Blots presented in the manuscript (set I)

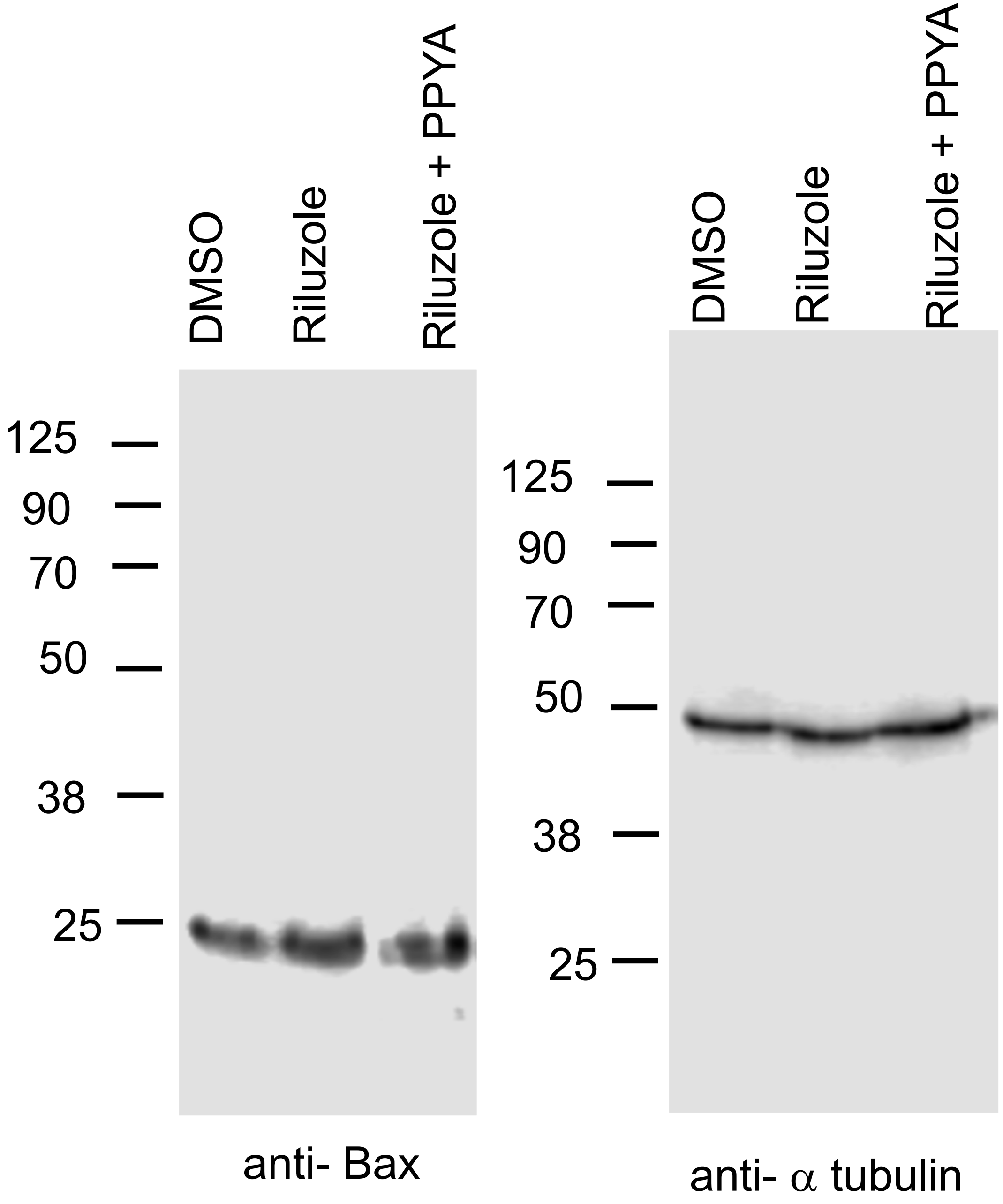

Figure 6A

Short exposure of blots presented in the manuscript (set I)

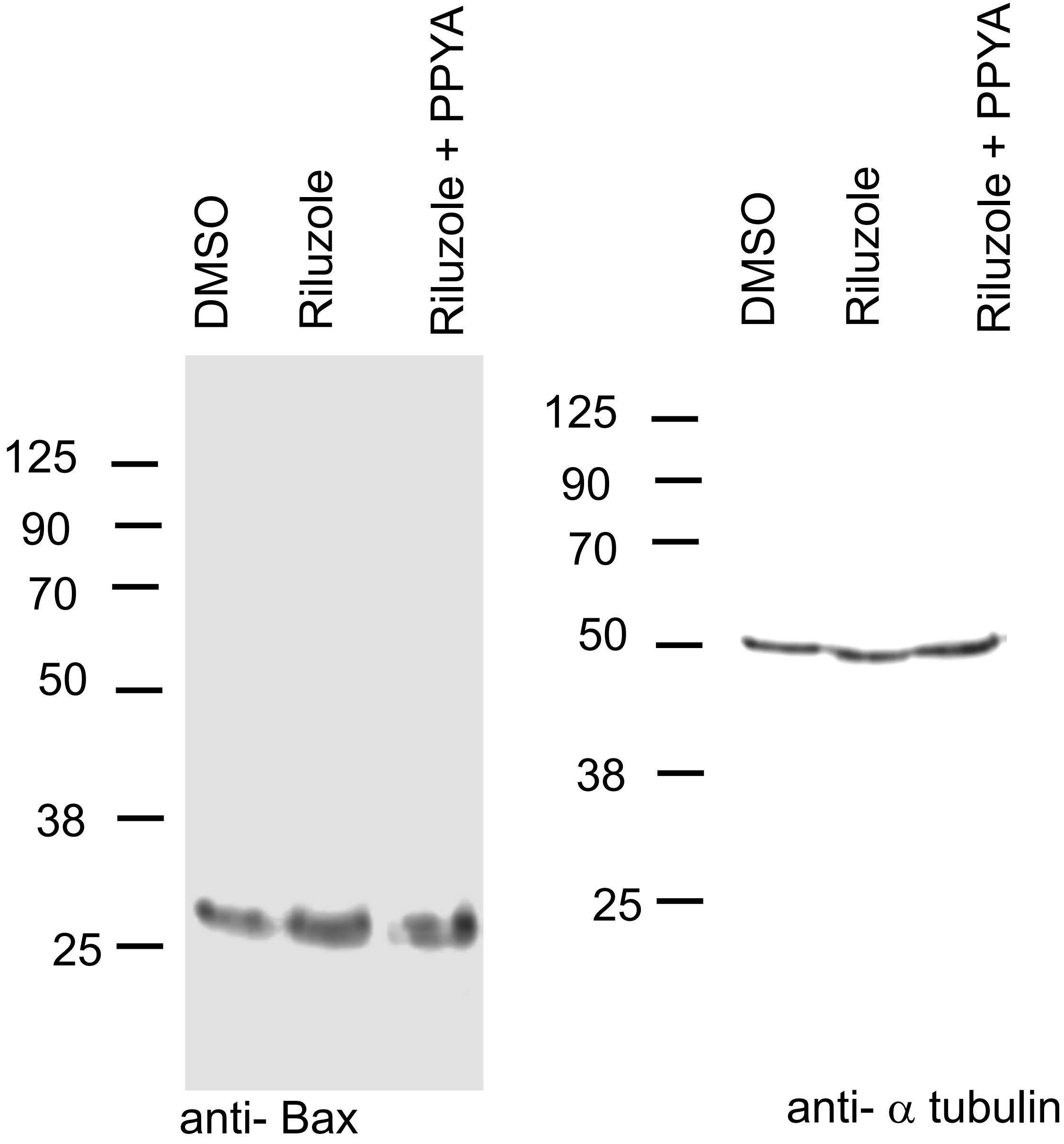

Short exposures Figure 6A

Replicate blots for Figure 6A (set II)

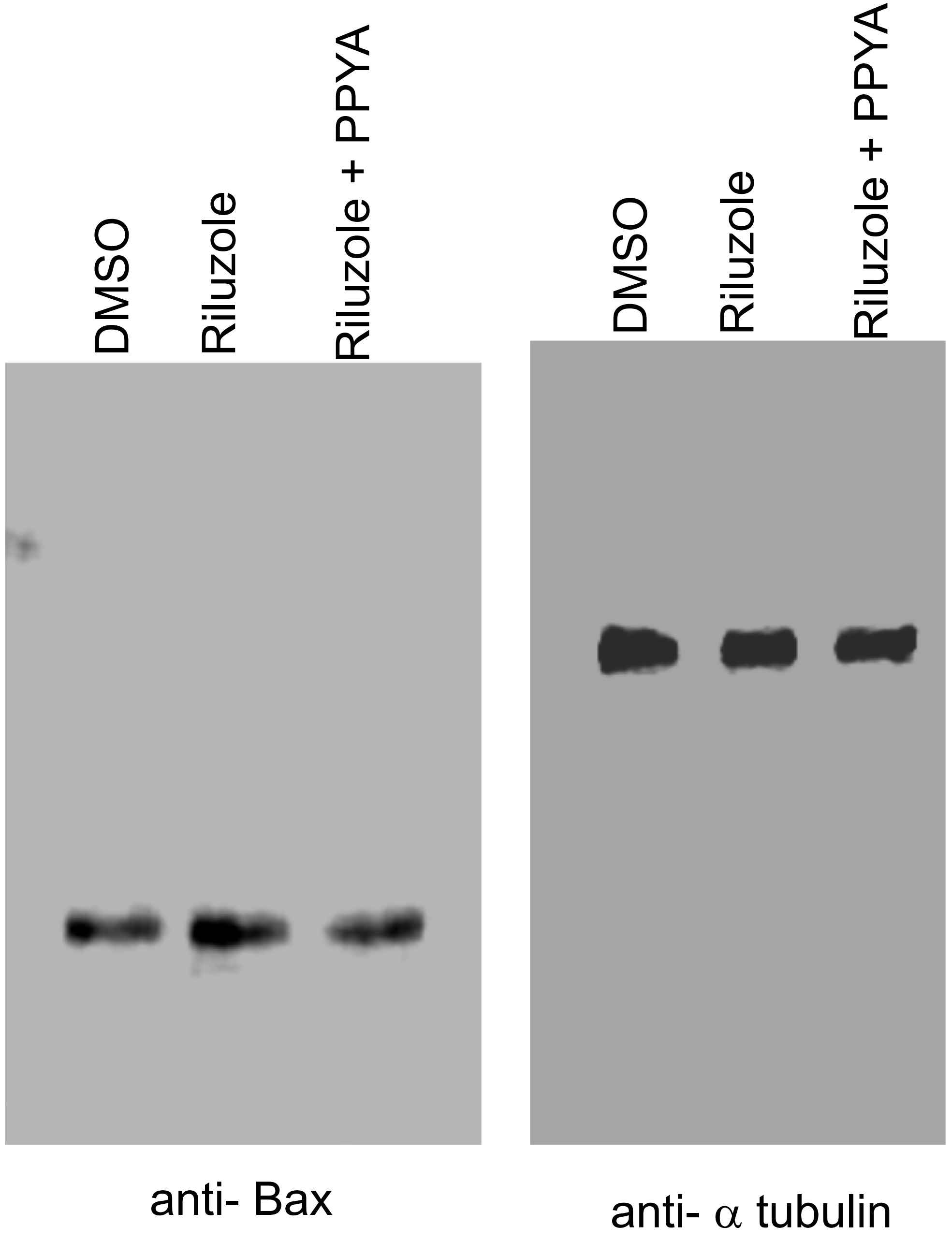

Low exposure of Replicate blots for Figure 6A (set II)

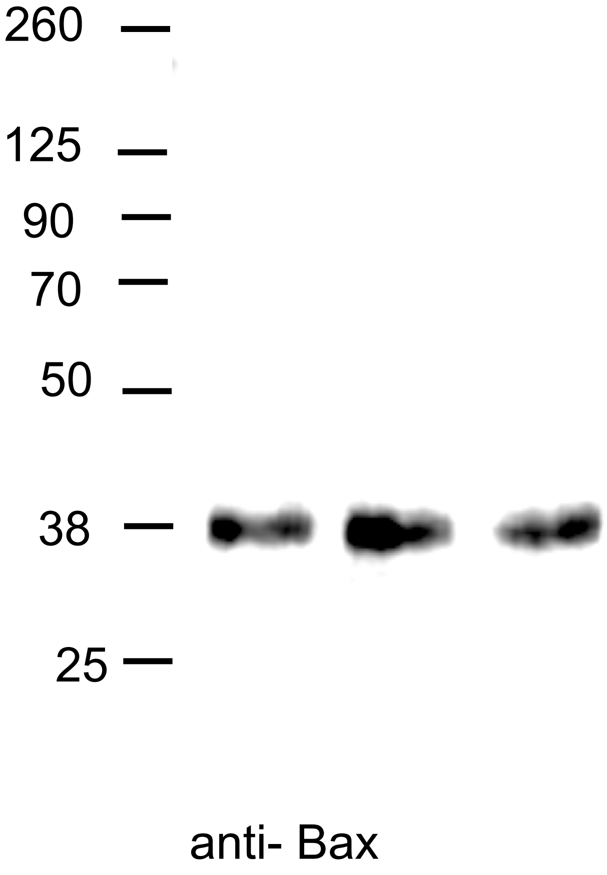

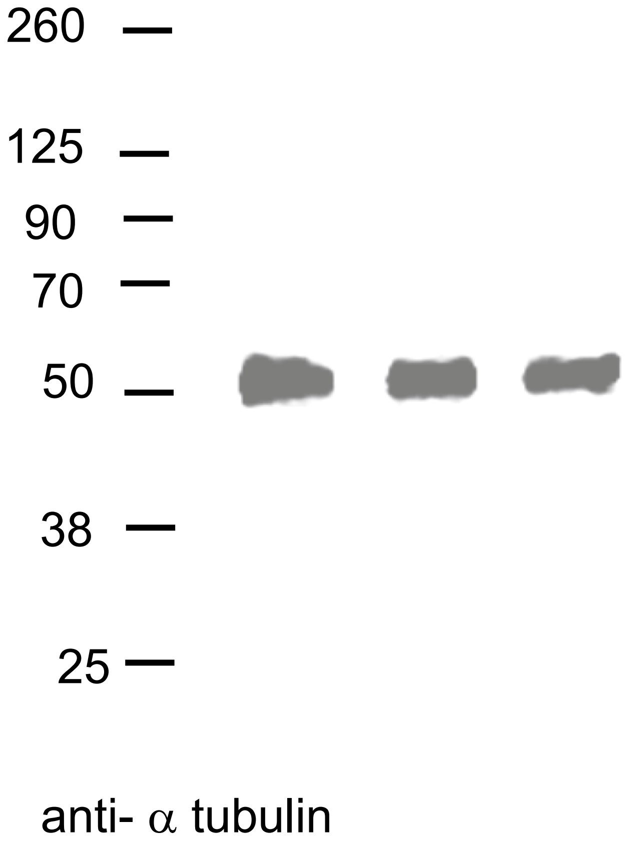

Supplement: Supplementary file 1 — Supplementary Information. [file 41598_2021_439_MOESM1_ESM.pdf]
